# Human Herpesvirus-6 Encephalitis

**DOI:** 10.5334/jbsr.2885

**Published:** 2022-10-10

**Authors:** Stijn Marcelis, Stephanie Vanden Bossche, Sven Dekeyzer

**Affiliations:** 1 UZA, BE; 2 U Gent, BE

**Keywords:** encephalitis, HHV-6, stem cell transplantation, MRI, HSE

## Abstract

**Teaching point:** HHV6 encephalitis is a specific complication to be considered in a patient with neurological symptoms after hematopoietic stem cell transplantation and typically shows symmetrical involvement of the limbic system.

## Case History

**Figure 1:**
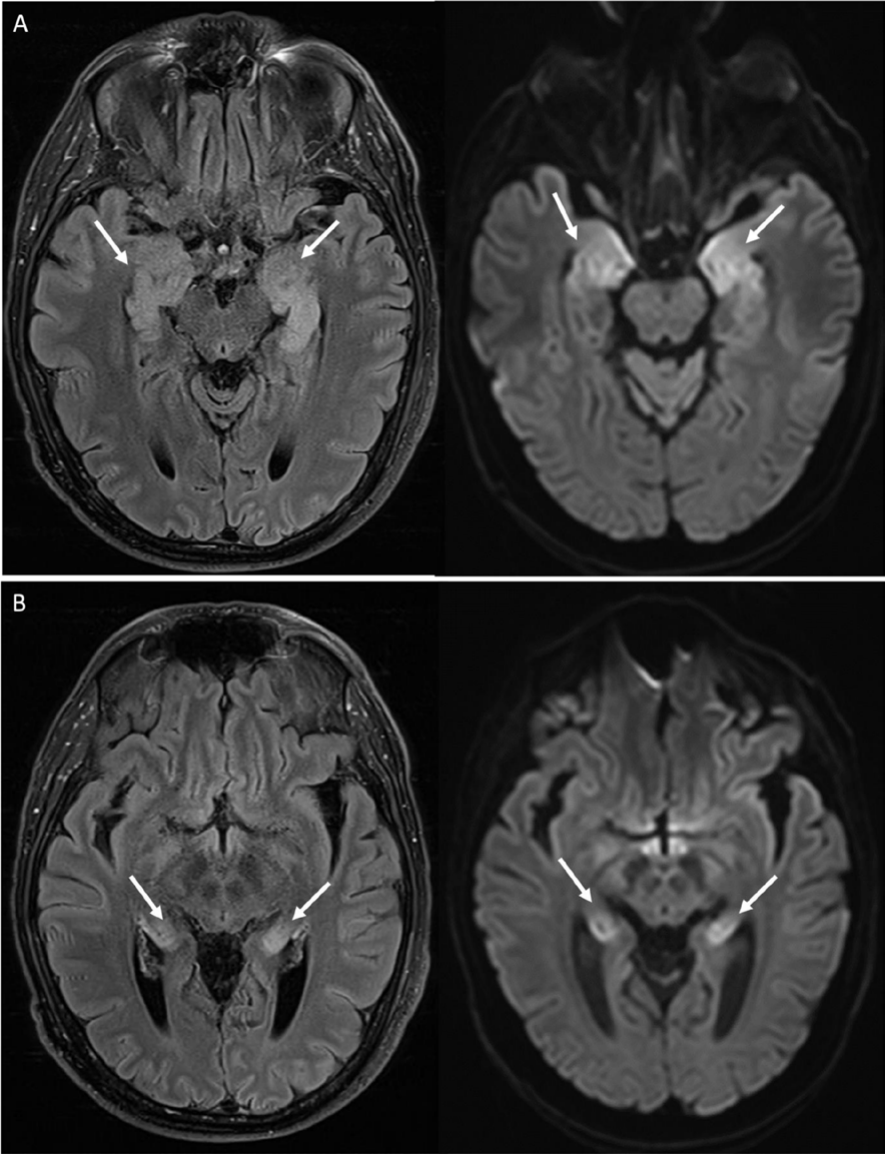


**Figure 2:**
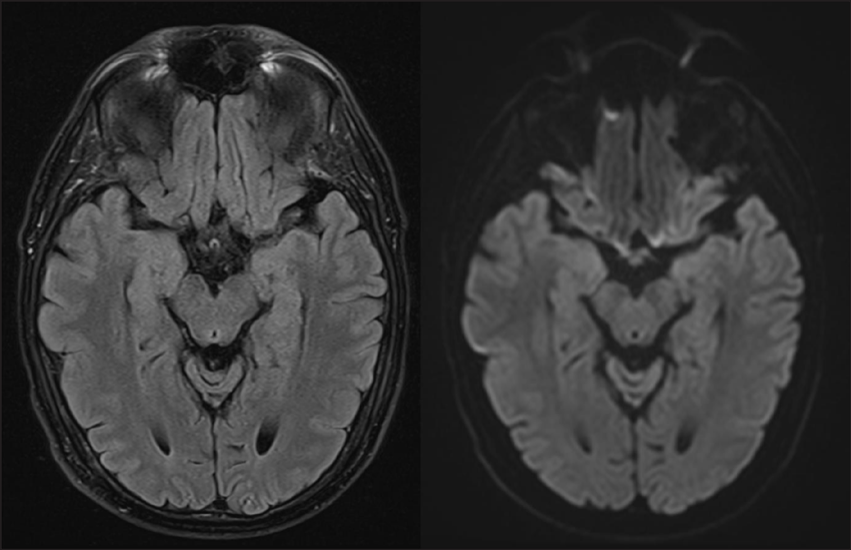


A 45-year-old man with myelodysplastic syndrome presented with confusion after allogeneic hematopoietic stem cell transplantation (HSCT). Magnetic resonance imaging (MRI) of the brain showed no abnormalities. Four days later he was admitted to the intensive care unit because of neurological deterioration. Electroencephalogram (EEG) showed epileptiform activity. The MRI examination was repeated and showed new bilateral symmetric T2 and FLAIR hyperintense signal in the limbic system (mesial temporal lobes, hippocampi, and amygdalae) suggestive of acute limbic encephalitis (Figure 1A–B, arrows). A lumbar puncture was performed and polymerase chain reaction (PCR) test for human herpesvirus 6 (HHV-6) was positive, thus a final diagnosis of post-transplant HHV-6 encephalitis was made. After treatment, a rapid and complete resolution of the imaging abnormalities was seen (Figure 2).

## Comment

HHV-6 is a neurotropic virus of the human herpes family and consists of two subtypes (type A and B). Around 90% of the population is seropositive for HHV-6 by the age of two. After primary infection, the virus remains latent in the body, and may reactivate in immunocompromised patients, especially after allogeneic HSCT. Reactivation of HHV-6 occurs in 30–70% of the patients and typically manifests two to four weeks after HSCT. Several complications may occur like graft-versus-host disease, bone marrow suppression or, as in our case, acute limbic encephalitis.

HHV-6-associated limbic encephalitis has clinical similarities with herpes simplex encephalitis (HSE) and presents with neurological symptoms such as amnesia, convulsions, and coma. On imaging, both HHV-6 encephalitis and HSE show an increased T2 and FLAIR signal and increased diffusion restriction bilaterally in the limbic system: the hippocampi and, variably, the adjacent mesial temporal structures (amygdalae and parahippocampal gyri). Contrast enhancement is usually absent in HHV-6 but can be seen in HSE. The extent and distribution of signal abnormalities is most useful to differentiate the two entities: HSE generally affects both the mesial and lateral temporal lobe (and commonly extratemporal regions) in an asymmetrical manner, whereas HHV-6 encephalitis is characterized by symmetrical signal changes limited to the mesial temporal lobes. The final diagnosis is made by a positive PCR test for HHV-6 in cerebrospinal fluid.

Differentiating HHV-6-associated encephalitis from HSE is important as HHV-6 is resistant to acyclovir which is the treatment of choice for HSE. HHV-6-associated encephalitis is treated with other antiviral medications like foscarnet or ganciclovir. HHV-6 responds significantly faster to treatment than HSE, with complete resolution of the abnormalities on MRI, in contrast to HSE that often shows irreversible damage [1].
